# Minimally Intrusive Optical Micro-Strain Sensing in Bulk Elastomer Using Embedded Fabry-Pérot Etalon

**DOI:** 10.3390/mi7040061

**Published:** 2016-04-06

**Authors:** Jungwook Paek, Qiang Li, In Ho Cho, Jaeyoun Kim

**Affiliations:** 1 Department of Electrical & Computer Engineering, Iowa State University, Ames, IA 50011, USA; paek@seas.upenn.edu (J.P.); shaunlee@iastate.edu (Q.L.); 2 Department of Civil, Construction & Environmental Engineering, Iowa State University, Ames, IA 50011, USA; icho@iastate.edu

**Keywords:** minimally intrusive, strain sensing, etalon, PDMS, soft MEMS

## Abstract

A variety of strain sensors have been developed to measure internal deformations of elastomeric structures. Strain sensors measuring extremely small mechanical strain, however, have not yet been reported due mainly to the inherently intrusive integration of the sensor with the test structure. In this work, we report the development of a minimally intrusive, highly sensitive mechanical strain transducer realized by monolithically embedding a Fabry-Pérot (FP) etalon into a poly(dimethylsiloxane) (PDMS) block test structure. Due to the extreme sensitivity of the FP resonance condition to the thickness of the spacer layer between the two reflectors, the limit of detection in the mechanical deformation can be as low as ~110 nm with a 632.8 nm laser used as the probing light. The compatibility of PDMS with additive fabrication turned out to be the most crucial enabling factor in the realization of the FP etalon-based strain transducer.

## 1. Introduction

Strain sensors, especially those embeddable inside the test structure, have been attracting intense research interest for their utility for structural health monitoring and bulk mechanical property measurement [1]. Since Thompson first reported the change in the electrical resistance of copper and iron bars due to their elongation in 1856, a variety of strain transduction mechanisms for metal and dielectric materials have been demonstrated. Most of them, however, are difficult to be embedded inside the test structure and often exhibit poor sensitivity at low strain levels after their embedding. A more fundamental issue, however, is the embedded strain sensors’ tendency to alter the host structures’ mechanical properties, which calls for new sensing schemes capable of measuring the strain level while minimally affecting the mechanical properties of the host structure. The recent advent of easily perturbable soft materials, such as elastomers, as the new building block of micromachines [2] and robots [3] is further heightening the need for such *minimally intrusive* strain sensing schemes.

In that respect, strain sensors based on nanostructures are highly promising because embedding them incurs little perturbation to the host structures’ mechanical properties. Examples include the strain sensors using graphene thin film and graphene woven fabrics [4], poly(dimethylsiloxane) (PDMS) and carbon nanotube composites [5], and fiber-optic Bragg gratings [6]. Of special interest are those supporting remote or wireless interrogation. It is a significant merit because realization of physical connection, such as electrical wiring, often perturbs the host structure’s mechanical properties even more seriously than does the sensor itself. In addition, physical connections severely complicate, if not disallow, multi-point strain sensing. To date, various electric, magnetic, and optical mechanisms have been adopted to realize such wireless interrogation. Among them, those based on optical effects have received particular attention owing to their spark-free, electromagnetic interference (EMI)-immune nature [7,8,9].

In this work, we report the development of a new embedded optical scheme for minimally intrusive strain sensing in a PDMS, a highly deformable elastomer, block. The main enabling factor is the *soft* Fabry-Pérot (FP) etalon fully and monolithically embedded within the PDMS test structure in a minimally intrusive fashion. The FP etalon consists of two extremely thin gold film reflectors separated by a thin layer of PDMS made of the same PDMS used to build the test structure. Such a minimally intrusive embedding is realized by exploiting the compatibility of PDMS with additive fabrication. The embedded strain sensor exhibits high sensitivity owing to the extreme dependence of the FP resonance condition on the thickness of the reflector separation. We validate our scheme by probing the deformation of a PDMS block with 110 nm limit of detection.

## 2. Principle of Operation

**Figure 1:**
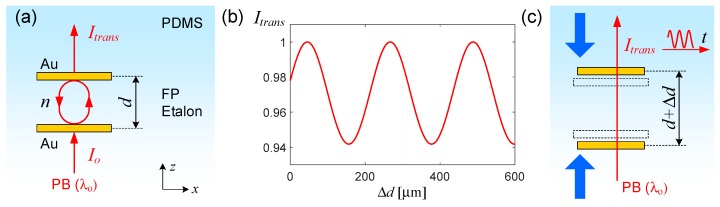
(**a**) A schematic illustration of the poly(dimethylsiloxane) (PDMS)-embedded Fabry-Pérot (FP) etalon. The probe beam (PB) with its wavelength λ_o_ impinges on the gold (Au) thin film reflector normally and undergoes FP resonance within the etalon with refractive index *n*; (**b**) Calculated output intensity *I_trans_* as a function of ∆*d* with *R* = 0.015, λ_o_ = 632.8 nm, *n* = 1.43); (**c**) Deforming (blue arrows) the etalon-containing PDMS block in *z*-direction will modify *d*. In turn, the resulting change in the resonance condition will modify *I_trans_*. Due to the narrow linewidth of PB, the output takes an amplitude-modulated form.

Figure 1a shows the basic configuration of the PDMS-embedded FP etalon. It consists of two reflective layers with nanoscale thickness, separated by a dielectric spacer with microscale thickness. The probe beam impinges normally on the etalon as a collimated beam. The FP resonance ensures only the frequency components with their *f* close to the FP resonance frequency *f_r_* = *m·c*_o_/(*n·*2*·d*), where *m*, *c*_o_, *n*, and *d* represent the integer order, the speed of light in vacuum, the refractive index of the resonator spacer material, and the mirror-to-mirror spacing, respectively, can pass through the etalon and emerge as the transmission with the intensity *I*_trans_ depending on *d* as:

*I*_trans_ ∝ (1 − *R*)^2^/[(1 − *R*)^2^ + 4*R·*sin^2^ (*kd*)] (1)

The etalon’s key enabling feature for strain sensing is its extreme dependence on the change in its mirror-to-mirror spacing *d* shown in Figure 1b. The sensitivity can be quantified as in [10]:

∂*I*_trans_/∂*d* ∝ −8*kR·*(1 − *R*)^2^·sin(*kd*)*·*cos (*kd*)/[(1 − *R*)^2^ + 4*R·*sin^2^ (*kd*)]^2^ (2)

In configurations with low *R* and *d* >> λ, ∂*I*_trans_/∂*d* ~ −4*kR·*sin(2*kd*) will produce low contrast but faithfully respond to Δ*d* sinusoidally. Based on this, we can turn the FP etalon into a transducer of mechanical strain by embedding it in an elastomeric volume as shown in Figure 1c. In this configuration, a *z*-direction deformation in the host structure will induce a corresponding change in *d*(Δ*d*) which, in turn, will modulate the resonance condition and shift the resonance frequency. If a narrow bandwidth laser light were used as the probe beam, the shift in the resonance frequency will translate into the modulation of the transmitted beam intensity, *I*_trans_. The sensitivity is so high that Δ*d* corresponding to only a quarter of the probe light’s wavelength in the resonator material can fully switch the transmission level from its maximum to minimum. For instance, in the case of a Helium-Neon (HeNe) laser (λ_o_ ~ 632.8 nm) probing a FP etalon loaded with PDMS (*n* ~ 1.43), Δ*d* ~ 110 nm induces a full swing between the minimum and maximum transmission levels. The optical output due to a microscale deformation will take amplitude-modulated forms, facilitating the estimation of deformation levels.

## 3. Fabrication

**Figure 2:**
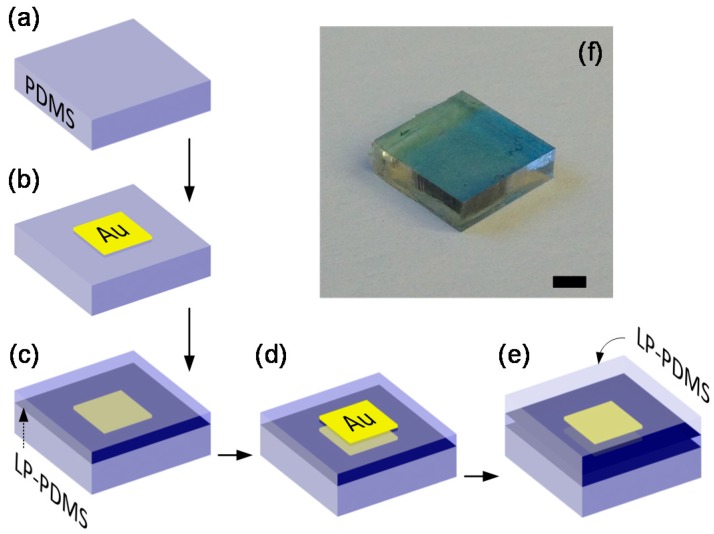
(**a–e**) Fabrication steps for PDMS-embedded soft FP etalon: (**a**) Preparation of a PDMS substrate with a pristine top surface; (**b**) Deposition of the Au mirror layer (Stenciled patterning was not used for this work but can be adopted for multi-point sensing); (**c**) Additive fabrication of the etalon’s spacer layer through spin-coating and curing of liquid phase (LP)-PDMS; (**d**) Patterning of the second mirror layer; (**e**) Total embedding through LP-PDMS overcasting; (**f**) Optical image of our final fabricated device (Scale bar: 3 mm).

Figure 2a–e shows the fabrication steps. We started out by preparing a PDMS substrate with a pristine top surface. To that end, a PDMS block was fabricated in a dust-free environment with minimal exposure to humidity. Cleaning with oxygen plasma was also performed to eliminate the contamination by organic materials. Then, we deposited a thin (~15 nm) gold (Au) layer through physical vapor deposition (PVD) to serve as the first reflector. Among various PVD techniques, we specifically chose sputtering mainly for its minimal thermal effect on the sample. We kept the deposition rate low, at ~10 nm per minute, so that the temperature on the sample side can be kept below the glass transition temperature of PDMS (~125 °C). Since this work mainly targets verifying the feasibility of the embedded FP etalon scheme for internal strain sensing, we deposited the gold layer over most of the PDMS block’s top surface. In future work targeting simultaneous strain sensing at multiple spots, we can also form an array of such reflectors by depositing the metallic material through a stencil. The completed Au/PDMS structure was again cleaned with oxygen plasma and kept in a sealed environment.

Then, we coated a thin layer of liquid-phase (LP)-PDMS on top surface of the Au/PDMS structure through spin-coating and then thermally cured it at 100 °C for an hour. At this stage, we confirmed that by paying adequate efforts to avoid contamination, we can still obtain coagulative bonding between the old and new PDMS layers even with a round of metal sputtering has been performed in between. With that confirmed, we proceeded to another round of gold sputtering to form the second reflector. Finally, the whole structure was immersed in LP-PDMS and thermally cured.

In the process described above, the most important enabling factor was the compatibility of PDMS with the additive fabrication [11]. It allows fabrication of complex structures at microscale by adding patterned PDMS blocks on a layer-by-layer basis, forming a new PDMS layer by solidifying LP-PDMS poured or spin-coated over the already completed PDMS layer, as shown in steps 3 and 5. As long as the top surface of the pre-existing PDMS layer is free of impurities and humidity, the two layers coagulate monolithically, causing no change in the mechanical properties of the final structure.

The optical micrograph in Figure 2f shows the completed structure. It is a FP etalon consisting of two ~15 nm-thick gold thin film reflectors separated by a ~15 µm-thick spin-coated PDMS layer and being embedded within a 3 mm-thick PDMS block. The slight blue tint comes from the wavelength-dependent absorption by the gold layer. We can reliably control the thickness values of the Au layer and the PDMS spacer layer by adjusting the sputtering and spin-coater parameters, respectively. In fact, the 15 nm Au layer thickness was close to the accuracy limit of the sputterer used for this work. For many applications, metallic layers thinner than 10~15 nm are not recommended due to the increasing level of granularity. The parallelism between the two Au layers hinges entirely on the flatness of the spin-coated PDMS spacer layer. It is a critical factor for the operation of regular, macroscale etalons but its impact diminishes in thin etalons such as ours. We conjecture that the two gold thin films, with their volume fraction at ~10^−5^, would impart little impact on the mechanical properties of the overall PDMS block, leading to a minimally intrusive strain measurement. Details of metallic nano-layer/elastomer composites, however, are scarce and demand further investigations.

## 4. Experimental Characterization and Analysis

**Figure 3:**
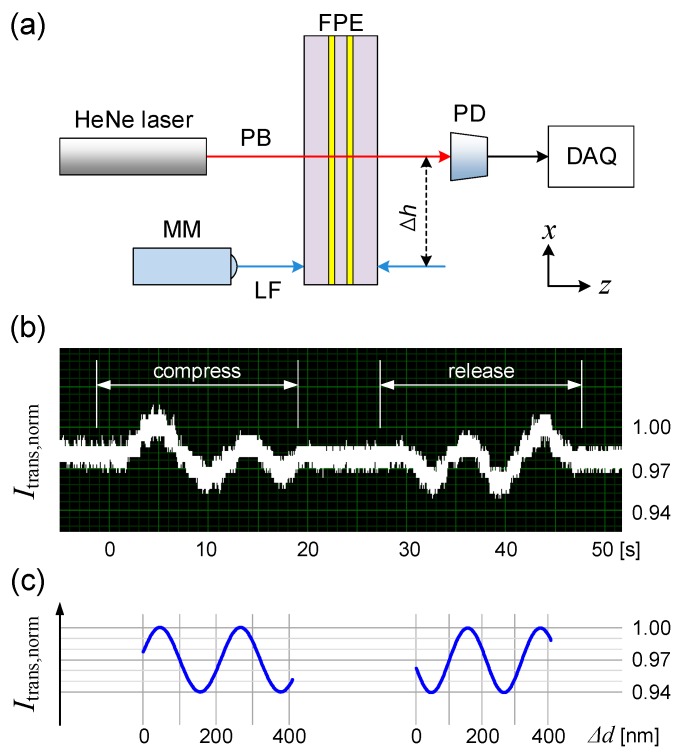
(**a**) A schematic diagram of the experimental setup for testing the strain sensing operation of the fully PDMS-embedded FP etalon (PB: Probe beam, FPE: Fabry-Pérot etalon, PD: Photodetector, MM: Micrometer, LF: Loaded force, DAQ: Data acquisition, ∆*h*: the separation between the optical probing and *z*-direction force application points); (**b**) The change in the probe beam’s transmission level (*I_trans,norm_*) due to the FPE’s *z*-direction deformation; (**c**) Calculated *I_trans,norm_* as a function of Δ*d*.

To validate the concept of the fully embedded FP etalon and assess the sensitivity of *I*_trans_ to the *z*-direction deformation of the hosting PDMS block, we tested the structure with our setup described in Figure 3a. A HeNe laser (λ_o_ ~ 632.8 nm, 0.8 mm beam diameter) was utilized as the probe beam. The intensity of the beam passing through the FP etalon was monitored with a photodetector which fed its data continuously to the home-made data acquisition system. To accurately deform the PDMS block, we employed a micrometer-controlled linear stage. The laser beam was aligned to pass through the center of the PDMS block while the micrometer was placed to apply force near its edge, located ~4 mm away. One cycle of deformation in this experiment consists of a 9 µm compression in *z*-direction followed by a total release over a 50-s temporal period. This small deformation level, amounting to only 0.3% of the total thickness, ensures that the FP response has arisen mainly from ∆*d* induced by compression, rather than bowing or tilt of the overall structure.

The values of *I*_trans_ measured over one cycle of externally induced deformation are plotted in Figure 3b. As expected from the monochromatic nature of the probe beam and its extremely narrow linewidth, the measured *I*_trans_ fluctuated periodically along the 9 µm *z*-direction compression, tracing a pattern similar to that of Figure 1b.

In fact, the generation of amplitude modulation is the most significant feature of the embedded FP etalon described above. It generates amplitude-modulated output signals and facilitates the estimation of the strain in comparison with other transduction schemes producing their outputs in the form of continuously changing analog signals. For example, the period count in Figure 3b indicates that the compression at the edge of the PDMS block had induced Δ*d* corresponding to 4 full swings between the minimum and maximum transmission levels, or ~440 nm, at the point of probing. A full release of the external force on the PDMS block produced an amplitude-modulated signal which exactly retraced the original signal from compression. Figure 3c shows the transmission levels calculated from Equation (1) as a function of Δ*d*. Aside from the noise and signal distortion due to the deformation process, the measured (Figure 3b) and calculated (Figure 3c) results exhibit excellent agreement, reaffirming the feasibility of embedded FP etalon-based micro-strain sensing. The measurement result turned out to be stable and repeatable throughout more than a 100 trials carried out over several days. The inherently amplitude-modulated nature of the output also suggests that the present system is better suited for sensing dynamic changes in strain occurring over a short period of time, during which the long-term variations in other parameters, such as the temperature or humidity, can be ignored.

The curve-fitting also revealed that the reflectance of the gold film reflector is ~0.015. We attribute the low reflectance of the Au reflector to the roughness of the PDMS substrate, granularity of the un-annealed Au layer, and its nanoscale thickness. In fact, we traded the reflectance with the ease of fabrication and minimally intrusive nature of the reflector. Despite the low reflectance, the etalon produced highly measurable modulation depth, which is promising for future applications.

Assuming that we can reliably resolve half a period, which is quite straightforward in Figure 3b, we can set the limit of detection at a quarter of the wavelength in PDMS, or ~110 nm, which is adequate for micro-strain sensing and future investigation of nanoscale soft-mechanics. In fact, the limit of detection can be further improved with the use of lasers with shorter wavelength as the probe beam. In addition, the amplitude-modulated output also indicates that the soft, mechanically tunable FP etalon can play the role of an extremely simple, nanoscale modulator of light intensity [9].

**Figure 4:**
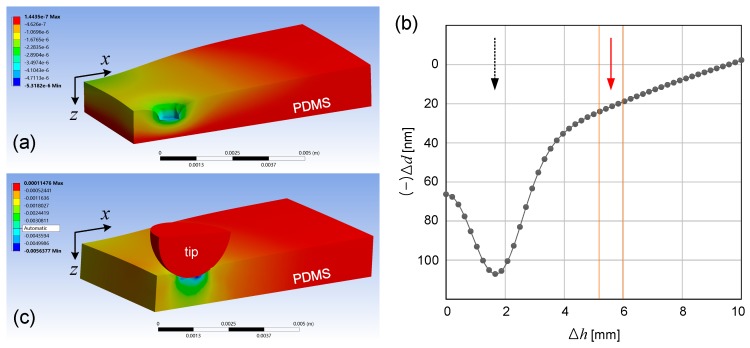
(**a**) Calculated *z*-direction deformation shape of the PDMS block. The tip of the micrometer was excluded for visual clarity; (**b**) Deformation in *z*-direction near the Au thin film. The black, dotted arrow and the red, straight arrow mark the points of force loading and optical probing, respectively. The two vertical lines represent the width of PB; (**c**) Calculated minimum principal elastic strain.

To qualitatively understand the deformation distribution of the PDMS block, we performed a nonlinear finite element analysis using ANSYS^TM^ (Ansys Inc., Pittsburgh, PA, USA). For PDMS material property, we adopted the experimentally measured Mooney-Rivlin hyperelastic model [12,13]. Based on the PDMS block’s structural symmetry, we analyzed a half of it. Owing to the physical contact between the soft hyperelastic PDMS and the rigid, hemispherical tip of the micrometer, the analysis involves severe geometric and material nonlinearities, for which we performed the large deformation contact analysis. Dynamic effects are ignored since the deformation was applied in a quasi-static manner. Figure 4a,b shows the simulated *z*-direction deformation shape and the cross-sectional distribution of *z*-direction deformation in the PDMS block near the gold thin film. Figure 4c shows the simulated principal elastic strain. As shown in Figure 4b, the ratio of the gold thin film’s *z*-direction deformation to the exerted displacement at Δ*h* ~ 4 mm is estimated to be ~20 nm, which is only ~5% of the experimentally measured 440 nm. We ascribe the discrepancy mainly to the severe nonlinearities in both the material characteristics of PDMS itself and its interaction with the spherically tipped, rigid micrometer. We consider such studies the natural and logical continuation of the current work. In fact, we expect that the experimental data accumulated through these embedded FP etalon-based measurements will greatly help refining the material and dynamic models for future computational studies.

## 5. Conclusions

In this work, we have demonstrated that it is feasible to realize a FP etalon inside an elastomer block in a totally embedded, monolithically integrated fashion and utilize it for minimally intrusive measurement of the elastomer structure’s internal strain in a wirelessly accessible format. We have described the steps for its fabrication in detail for future adoptions. The most crucial enabling factor turned out to be the compatibility of PDMS with additive fabrication, which allowed layer-by-layer realization of complex, multi-material composite structures such as the metallic film-based FP etalon. The characterization results also confirmed that the embedded FP etalon can transduce the externally applied strain into optical signals in amplitude-modulated format, which facilitates the measurements and processing tremendously. Thanks to the inherent sensitivity of the FP etalon, the embedded transduction scheme exhibited a submicron-scale limit of detection at ~110 nm. To the best of our knowledge, this is the first demonstration of a FP etalon functioning as a micro-strain transducer fully and monolithically embedded within an elastomeric volume. The results are promising for future applications of minimally intrusive strain sensing. One direct route to further improve the detection accuracy is to increase the reflectance of the Au layers through optimization of the fabrication process. Refinement of the computational model of the system through further material characterization is another task to be followed up.
